# Registry-based stroke research in Taiwan: past and future

**DOI:** 10.4178/epih.e2018004

**Published:** 2018-02-04

**Authors:** Cheng-Yang Hsieh, Darren Philbert Wu, Sheng-Feng Sung

**Affiliations:** 1 Department of Neurology, Tainan Sin Lau Hospital, Tainan, Taiwan; 2 School of Pharmacy, Institute of Clinical Pharmacy and Pharmaceutical Sciences, National Cheng Kung University College of Medicine, Tainan, Taiwan; 3 Division of Neurology, Department of Internal Medicine, Ditmanson Medical Foundation Chiayi Christian Hospital, Chiayi City, Taiwan; 4 Department of Nursing, Min-Hwei Junior College of Health Care Management, Tainan, Taiwan

**Keywords:** Database, Electronic health records, Medical record linkage, Registries, Research, Stroke

## Abstract

Stroke registries are observational databases focusing on the clinical information and outcomes of stroke patients. They play an important role in the cycle of quality improvement. Registry data are collected from real-world experiences of stroke care and are suitable for measuring quality of care. By exposing inadequacies in performance measures of stroke care, research from stroke registries has changed how we manage stroke patients in Taiwan. With the success of various quality improvement campaigns, mortality from stroke and recurrence of stroke have decreased in the past decade. After the implementation of a nationwide stroke registry, researchers have been creatively expanding how they use and collect registry data for research. Through the use of the nationwide stroke registry as a common data model, researchers from many hospitals have built their own stroke registries with extended data elements to meet the needs of research. In collaboration with information technology professionals, stroke registry systems have changed from web-based, manual submission systems to automated fill-in systems in some hospitals. Furthermore, record linkage between stroke registries and administrative claims databases or other existing databases has widened the utility of registry data in research. Using stroke registry data as the reference standard, researchers have validated several algorithms for ascertaining the diagnosis of stroke and its risk factors from claims data, and have also developed a claims-based index to estimate stroke severity. By making better use of registry data, we believe that we will provide better care to patients with stroke.

## INTRODUCTION

As has been observed in other developed countries, Taiwan has experienced a significant transition in the epidemiology of stroke [1]. Taking ischemic stroke as an example, the age-standardized incidence of first-ever ischemic stroke has declined from 142.3 to 129.5 per 100,000 adults between 2000 and 2011 [2]. The 1-year recurrence rate of stroke among patients with first-ever ischemic stroke decreased even more steeply from 9.6 to 7.8% during this period [2]. Improved identification and control of vascular risk factors, as well as adequate secondary prevention, may have contributed to the epidemiological trends of stroke in Taiwan [2,3].

Furthermore, the quality of stroke care, as represented by adherence to performance measures for acute ischemic stroke, also improved from 2000 to 2012 [3]. In particular, the rate of thrombolytic therapy for acute ischemic stroke and the rate of door-to-needle time within 60 minutes both significantly increased [4]. Meanwhile, in recent years, stroke has moved from the second to the fourth leading cause of death in Taiwan [5]. Something must have been done right for such progress to have been made, and we speculate that the development of stroke registries and the associated quality improvement initiatives likely played an important role in this progress. In this article, we review what we have learned about stroke registries over the past decade. Specifically, we focus on how researchers have used registry data to conduct stroke research, including data sharing with a common data model and linkage between registry data and administrative claims data.

## WHAT IS A STROKE REGISTRY?

A clinical registry is an observational database, usually focusing on a clinical condition, procedure, therapy, or population. A stroke registry can be defined as “an organized system for the collection, storage, retrieval, analysis, and dissemination of information on individual patients who have had a stroke” [6].

An ideal stroke registry is nationwide and enrolls patients from as many participating hospitals as possible in order to increase representativeness and avoid selection bias [7]. For example, the Riks-Stroke register in Sweden, launched in 1994, has covered all hospitals that admit acute stroke patients across the country since 1998 [8]. Appropriate data structure and governance policies are needed to keep a nationwide stroke registry sustainable and operating well. Through the publication and communication of results, a stroke registry should be helpful for improvement of stroke care quality, health policy, and the outcomes of patients [7].

## STROKE REGISTRIES AND THE CYCLE OF QUALITY IMPROVEMENT

**Figure 1.**
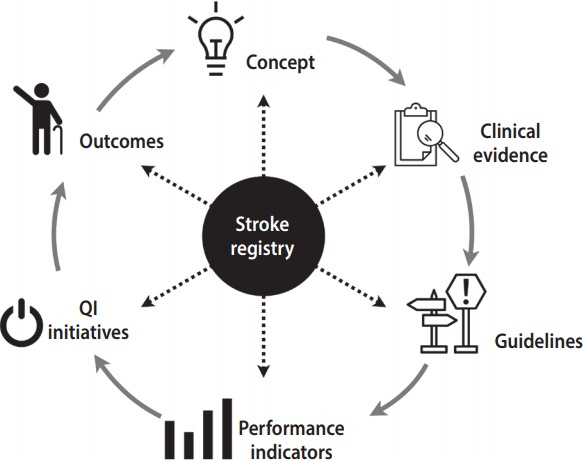
The role of the stroke registry in the cycle of quality improvement. Adapted from Bhatt et al. J Am Coll Cardiol 2015;66:2230-2245 [6]; Califf et al. J Am Coll Cardiol 2002;40:1895-1901 [9].

Clinical registries are at the center of the cycle of quality improvement in patient care [6,9]. Stroke registries are no exception (Figure 1). For example, the Taiwan Thrombolytic Therapy for Acute Ischemic Stroke (TTT-AIS) register is a therapy-specific stroke registry, aiming at monitoring the safety and effectiveness of recombinant tissue-type plasminogen activator (rt-PA) in Taiwanese stroke patients [10]. Although rt-PA was approved for patients with acute ischemic stroke in Taiwan in 2002, no placebo-controlled, randomized clinical trials have been conducted on the use of rt-PA for acute ischemic stroke in Taiwan or other Asian countries. Concerns remain regarding the safety and effectiveness of rt-PA in Taiwanese patients, for whom the risk of bleeding, including potentially lethal symptomatic intracranial hemorrhage (SICH), may be higher than for Western patients. These concerns lay behind the rationale and concept of the development of the TTT-AIS register.

The TTT-AIS register was a centralized and web-based registration system that collected patient data from 23 major hospitals in Taiwan. The first phase of the registry project took place from 2004 through 2008 [10], and the second phase was extended to 2011 [11]. The most salient finding from the TTT-AIS is the interaction between age and rt-PA dose. That is, in patients aged greater than 70 years, a lower dose of rt-PA was associated with less SICH and better functional outcomes [11]. This experience underscores the importance of clinical registries in the cycle of quality improvement. Using registry data, we have turned a concept into clinical evidence, and developed our guidelines accordingly. Additionally, of course, evidence from observational studies should be further examined in randomized controlled trials [12].

## TAIWAN STROKE REGISTRY

Another example illustrating the role of registry-based research in the cycle of quality improvement for stroke care is the Taiwan Stroke Registry (TSR). The TSR is our first large nationwide stroke registry; it was launched in 2006 and is still ongoing. It is also centralized and web-based. The TSR enrolls patients who are admitted within 10 days of stroke onset because of 1 of the 4 major types of stroke (i.e., ischemic stroke, intracerebral hemorrhage, subarachnoid hemorrhage, and transient ischemic attack), and follows them for 6 months afterward. During the early phase of the TSR between 2006 and 2008, a total of 39 hospitals across the country participated in the registration project. Most of them are tertiary referral medical centers and regional hospitals. Data were collected prospectively and entered by TSR-trained study nurses. The TSR employed several quality assurance processes to ensure data quality. About 18% of the admitted stroke patients in Taiwan were registered during this period [13]. More than 100,000 stroke events have been recorded in the TSR up to 2015 [14]. Nevertheless, the TSR will be more representative of the general population if more hospitals are recruited into the TSR, particularly district hospitals.

The first paper from the TSR was published in *Circulation* in 2010 [13]. From the title of this paper, we can see the ambitions of the project, which was envisioned to be the “Get With The Guidelines-Stroke” program in Taiwan, the Taiwanese version of the American Heart Association/American Stroke Association Get With The Guidelines-Stroke program. The data presented in that paper documented inadequacies in several performance measures, such as rt-PA for patients with ischemic stroke presenting within 2 hours of onset, anticoagulation for patients with ischemic stroke or transient ischemic attack with atrial fibrillation, and lipid-lowering therapy for patients with ischemic stroke or transient ischemic attack with low density lipoprotein-cholesterol ≥ 100 mg/ dL [13].

The value of this paper is that it disclosed how we performed on several quality indicators developed from evidence-based guidelines [15]. To address these issues, the Taiwan Joint Commission on Hospital Accreditation undertook an initiative seeking to improve the quality of acute stroke care by adopting the Breakthrough Series (BTS) model [16]. Hospitals interested in improving the quality of acute stroke care were encouraged to participate in the BTS-Stroke activity. A total of 24 major hospitals across the country joined that quality improvement activity during 2010 and 2011. The BTS-Stroke activity consisted of 3 learning sessions and a final summative meeting throughout a 1-year period. Each hospital had a BTS team made up of 3 to 7 core members. Key elements of the BTS activity included topic selection, faculty recruitment, enrollment of participating organizations and teams, learning sessions, action periods, improvement of the model, and outcome measurement and evaluation [16]. With the implementation of this activity, we have observed significant trends of increasing adherence to stroke performance measures and decreasing rates of recurrent stroke and mortality in patients with ischemic stroke in Taiwan [3,16].

## RESEARCH FROM THE TAIWAN STROKE REGISTRY

Although the raw data from the TSR are not open to researchers, site investigators from all participating hospitals can submit their study proposals to the research committee of the TSR. Once study proposals are accepted, staff of the central data laboratory analyze the data and send back the results to investigators for drafting the manuscript. Several research articles from the TSR have been published in prestigious journals, mainly focusing on the predictors of patient-centered outcomes [17-20]. For example, Tang et al. [18] found that low pulse pressure upon admission was associated with poorer 3-month outcomes in patients with acute ischemic stroke. The findings from these studies may provide useful information for the prognostication and management of stroke patients.

However, several limitations of the TSR should be addressed. Because of the scale of the registry, adequate funding is required for its operation and maintenance. Some trade-offs between the comprehensiveness of its data elements and the cost of obtaining quality data are thus inevitable. A few clinically relevant pieces of data are not collected, such as the dose of rt-PA administered, post-thrombolytic SICH per various definitions, and the pre-stroke modified Rankin Scale score. Missing data are another common problem for such a registry that tries to keep track of large numbers of patients. Because of shortages of funding and human resources, obtaining follow-up data may be difficult if stroke patients seek medical services in hospitals other than the initial hospital of care and do not come back for follow-up. Furthermore, patients with missing data may have either recovered so well that follow-up is perceived to be unnecessary or have been placed in a nursing home due to poor functional status, making them unable to return for follow-up. According to a study from the TSR, approximately one-third of the study patients had missing information on 3-month outcomes [18]. Biases may occur if a considerable proportion of data are missing non-randomly.

## TAIWAN STROKE REGISTRY AS A COMMON DATA MODEL

To minimize these problems, enthusiastic researchers from some participating hospitals have tried to combine their data and add more data elements to the existing data structure of the TSR as a workaround. The contribution of the TSR in this regard cannot be overlooked because, by participating in the TSR, many hospitals have built their own stroke registries on top of the common data model set up by the TSR [21]. Despite being based on a much smaller patient pool, several papers drawing on those registries have been published in high-impact journals [22-24]. Lin et al. [22] investigated the readmission risk, causes, and risk factors after discharge in patients with acute stroke. Chen et al. [23] observed that intravenous thrombolysis with standard-dose rt-PA might have a similar profile of safety and effectiveness to that of intravenous thrombolysis with low-dose rt-PA in Taiwanese patients. Sung et al. [24] compared and externally validated several SICH risk-scoring systems, which could help assess the risk of post-thrombolysis SICH in patients with acute ischemic stroke. These studies were all intended to answer practical questions about patient care.

## STROKE REGISTRIES AND ELECTRONIC MEDICAL RECORDS

Apart from the TSR, another large stroke registry worth mentioning is the Stroke Registry in Chang Gung Healthcare System (SRICHS). The Chang Gung Medical System (CGMS) is among the largest healthcare groups worldwide, containing 10,000 beds, 8.2 million outpatient visits per year, and more than 3,000 physicians in a network of 7 branch hospitals across the country. It is estimated that a third of the Taiwanese population have sought treatment from the CGMS [25]. More than 4,000 patients with ischemic stroke were admitted to its branch hospitals in a single year [26]. Instead of participating in the TSR, stroke teams in the CGMS developed their own electronic chart-based stroke registry system in 2006, known as the SRICHS [27]. It was innovative at the time it was created because it was the first automated electronic chart-based stroke registry in Taiwan. Stroke patients are automatically enrolled into the SRICHS based on the International Classification of Disease codes at the initial patient encounter. In addition, its data collection mechanism is embedded within the hospital information system. Most of the data elements are automatically filled in with data downloaded from the hospital information system, or manually entered using pull-down menus during the process of medical record writing [27]. Therefore, the SRICHS is more time- and labor-efficient than the TSR. The registration system has been adopted by other medical specialties within the CGMS [28]. Several interesting studies have been published by the SRICHS group [29-31]. The main shortcoming is that the registration system is currently only operable inside the CGMS.

## RECORD LINKAGE

Taiwan’s National Health Insurance Research Database (NHIRD), derived from the claims data of Taiwan’s National Health Insurance, is widely used in various kinds of clinical research, including studies of risk factors of diseases, outcomes research, healthcare utilization, and patterns of drug prescription [32]. As compared with clinical registries, nationwide administrative claims databases like the NHIRD have several advantages. They generally have a large sample size, contain serial longitudinal data, and are representative of the entire population. They are thus particularly suitable for longitudinal outcome assessment [33].

According to a PubMed search, as of December 2017, more than 200 stroke-related research articles have been published based on data from the NHIRD. However, administrative claims data are collected for insurance reimbursement rather than for the conduct of research. Therefore, data elements in the NHIRD should be properly validated before they can be used in research. Most importantly, the NHIRD lacks information about stroke severity, which is a critical piece of clinical information regarding patients with stroke. Stroke severity varies greatly among patients and largely determines patient outcomes [34]. Consequently, the validity of research findings from claims-based stroke studies might be undermined.

Through record linkage between clinical and claims data using indirect personal identifiers [35], researchers have been trying to find solutions to the problems described above. Cheng et al. [36] and Hsieh et al. [37] separately validated the diagnosis of ischemic stroke in the NHIRD and found that their algorithms could identify cases of ischemic stroke in the NHIRD with a sensitivity of 94.5 to 97.3% and a positive predictive value of 88.4 to 97.8%. Using hospital records as the reference standard, Cheng et al. [38] established algorithms to verify the mortality status of patients with ischemic stroke in the NHIRD. By linking stroke registry data to the NHIRD, Hanberg et al. [39] evaluated and validated various algorithms to ascertain stroke risk factors in patients with ischemic stroke, transient ischemic attack, or intracerebral hemorrhage. Moreover, using the same record linkage method, Sung et al. [40] developed a claims-based stroke severity index that can be used to estimate the National Institutes of Health Stroke Scale score in patients with ischemic stroke in the NHIRD. This stroke severity index has been separately validated in patients with ischemic stroke [41] and intracerebral hemorrhage [42] and has been applied in several studies of stroke outcomes that have been published in leading journals [43-45].

## FUTURE PERSPECTIVES

**Figure 2.**
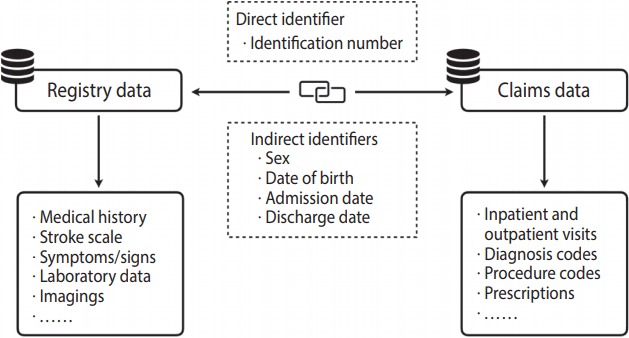
Linkage between registry and claims data using direct or indirect identifiers.

While on the one hand, the validated methods for using stroke registry data as the reference standard help corroborate the findings of stroke research based on the NHIRD, record linkage with claims databases may, on the other hand, give a further boost to research based on stroke registry data. Ideally, registry databases with rich and detailed clinical information, once linked with claims databases with long-term outcome data, could offer valuable opportunities for outcomes research (Figure 2). In this way, the strengths of both types of databases can be integrated [33]. For example, despite being applied to a clinical condition other than stroke, record linkage between a clinical registry of patients undergoing 24-hour Holter monitoring and the NHIRD has produced fruitful research results [46,47]. In addition, record linkage will enable us to determine the differences in the baseline characteristics and outcomes between stroke patients admitted to hospitals that do and do not participate in the stroke registry [48], and thus provide an opportunity to examine the generalizability of the results of studies that use registry data. Therefore, linking large-scale stroke registry data to the NHIRD appears to be a promising direction for future registry-based stroke research.

Even though record linkage using multiple indirect personal identifiers is feasible [35], the use of a direct personal identifier (e.g., personal identification numbers) is the gold standard for linking datasets (Figure 2). Record linkage between the NHIRD and other national databases, such as the death registry and birth registry, as well as clinical registry databases using personal identification numbers, has long been made available within the Data Science Center of the Ministry of Health and Welfare located in Taipei. Recently, with advances in network security, researchers have had the opportunity to access and link these national databases via a virtual private network from local branch offices of the Data Science Center across the country. However, many obstacles still lie ahead, including the public’s concern for privacy, protests from human rights organizations against the collection and use of health and welfare data, and the amount of red tape necessary to get permission to link data.

In this regard, the Registry of Canadian Stroke Network (RCSN) is a useful example. Initially, the registry enrolled only patients who had given written informed consent because of the concern that privacy legislation may mandate informed consent for entering patients into clinical registries. They soon found that, in addition to increasing expenses, obtaining written informed consent led to major selection biases, thus threatening the validity of the registry data [49]. Therefore, under new health privacy legislation in Ontario, Canada, the RCSN was designated as a “prescribed registry,” which permitted the RCSN to operate the registry without consent from patients [50]. The RCSN also stopped conducting follow-up interviews with the patients. Instead, patient outcomes are traced by record linkage between the RCSN database and population-based administrative databases using unique encrypted identifiers under the provincial law [50]. Such record linkage may provide a potential remedy for missing data during follow-up.

The successful experience of implementing an electronic chart-based stroke registry system by the SRICHS also inspires us to imagine a broader coverage of data elements in a stroke registry. In the era of big data and the Internet of Things, numerous physiological parameters can be automatically recorded and imported into registry databases. Furthermore, even unstructured medical records can be processed using natural language processing tools to generate useful information [51]. The future development of stroke registries will surely require collaboration between stroke researchers and information technology professionals [52].

Finally, we should seek opportunities to assemble a multinational network of stroke registries in the future. In addition to large sample sizes, multinational stroke registries enable cross-country comparisons and the generalization of study results [14]. For example, similar to that in Taiwan, the healthcare system in South Korea provides coverage to all its citizens. With the aim of describing stroke statistics and the quality of stroke care, a web-based, prospective stroke registry, the Clinical Research Center for Stroke–Fifth Division stroke registry, was launched in 2008 and has been maintained with high quality [53,54]. Although registry databases in different countries may be heterogeneous in a variety of aspects, a distributed network approach can be applied [55]. In this approach, databases can be integrated through a common data model without sacrificing the operational independence of individual stroke registries [14], even though we may face challenges in the harmonization of data elements across registries.

## CONCLUSION

Over the past decade, registry-based stroke research and associated quality improvement campaigns have enhanced the quality of care for stroke patients in Taiwan. Record linkage between stroke registries and administrative claims databases has made a significant contribution to claims-based studies by providing methods for ascertaining the diagnosis of stroke and its risk factors, as well as estimating stroke severity, from claims data. In the future, many further possibilities are available if we can make better use of the data we have registered.
